# The Role of Partnerships in Supporting COVID-19 Vaccine Uptake Among Migrants: A Qualitative Case Study from Tamil Nadu and Punjab, India

**DOI:** 10.3390/vaccines13010062

**Published:** 2025-01-12

**Authors:** Ankita Meghani, Bharathi Palanisamy, Sunita Singh, Tanya Singh, Natasha Kanagat, Anil Gupta, Kapil Singh, Gopal Krishna Soni

**Affiliations:** 1 PATH USA, Seattle, WA 98102, USA; 2 John Snow India Pvt. Ltd., Delhi 110070, India; bharathi.vmp@gmail.com (B.P.); singhsunita10@hotmail.com (S.S.); tanya.25@gmail.com (T.S.); gupta.dranil@gmail.com (A.G.); gopal_soni@in.jsi.com (G.K.S.); 3 SRM Institute of Science and Technology, Kattankulathur 603203, Tamil Nadu, India; 4 JSI Research and Training Institute USA, Arlington, VA 22202, USA; natasha_kanagat@jsi.com; 5 UNICEF, New Delhi 110011, India; drkapil.mohfw@gmail.com

**Keywords:** COVID-19, vaccine uptake, migrants, strategies, behavioral and social drivers framework, influencers, partnerships, qualitative case study, faith-based organizations

## Abstract

Background: During the COVID-19 pandemic, migrant populations remained under-immunized due to limited access to health care, language barriers, and vaccine hesitancy. The USAID-funded MOMENTUM Routine Immunization Transformation and Equity project supported the government in collaborating with various local health and non-health partners to identify and vaccinate migrants. This case study examines the roles of project partners and the strategies each entity implemented to increase COVID-19 vaccine uptake among migrants, as well as the perceptions regarding the effectiveness of these strategies. Methods: We designed a qualitative explanatory case study guided by the Behavioral and Social Drivers framework and RE-AIM implementation science frameworks. We conducted 31 focus group discussions and 50 in-depth interviews with migrants, project partners, community leaders, and government stakeholders in Tamil Nadu and Punjab. Results: In both states, partnerships with health departments, private employers, and community-based organizations were essential for identifying and vaccinating un- and under-vaccinated migrant groups. In Tamil Nadu, collaboration with the Department of Labor and mobile medical units facilitated vaccination camps at construction sites. In Punjab, religious institutions organized sessions at places of worship, and the Border Security Force enabled health workers to reach migrants living near the border. In both states, key strategies—involving influencers to discuss the importance of vaccine safety and value, bringing vaccination services to migrants’ workplaces and homes at flexible times and mandating workplace vaccination to encourage vaccination—shifted perceptions towards vaccination and increased vaccine uptake among migrants. Conclusions: The strategies and partnerships identified in this study highlight the broader implications for future public health interventions, demonstrating that collaboration with the private sector and faith-based organizations can enhance routine immunization efforts, particularly when localized to organizations that understand community needs and can address specific barriers and motivators.

## 1. Introduction

Migrants make up 41.4 million of India’s 474 million workers—roughly 9% of the total workforce according to the most recent Census data [1,2,3]. They are predominantly from economically disadvantaged states [4] and often travel to rapidly urbanizing city centers such as in Delhi, Tamil Nadu, Punjab, Maharashtra, Gujarat, Andhra Pradesh, and Kerala in search of better work opportunities [5]. Once there, they frequently find work in unorganized or informal sectors, such as brick kilns, construction, and long-distance trucking.

Poor working conditions in these fields heightened their risk of COVID-19 exposure and increased the likelihood of virus transmission when compared to the general population [6,7,8]. This situation was exacerbated by the national lockdown due to COVID-19, which forced 11.4 million migrants to return to their states of origin [9]. Those who were stranded in the cities to which they had migrated were stigmatized as SARS-CoV-2 virus carriers [10].

Amidst the evolving pandemic landscape, those who did not return to their states of origin or remained in their current work locations/residences faced significant hurdles accessing essential social security programs and health care, which were tied to regional entitlements programs. Ration cards, the official documents that allow access to these programs, were not valid across state lines [8]. A 2020 survey of migrant communities—largely from Madhya Pradesh and Uttar Pradesh working in construction, agriculture, and manufacturing—found that 42% of daily wage workers did not have rations or food for the next day; roughly 33% had no money to buy rations; 14% had no ration card; and 12% had ration cards but could not access the public food distribution system because of their migrant status [11]. Furthermore, language barriers, limited knowledge of and unfamiliarity with the new state’s health system, and fear of losing wages reduced health-seeking behavior among migrants and limited their access to high-quality care, including COVID-19 vaccines [12].

**Figure 1:**
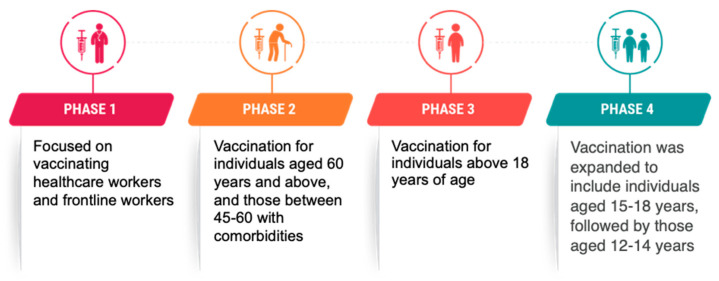
India’s approach to COVID-19 vaccine administration [19,20,21,22,23,24].

Despite India’s phased approach, prioritizing special groups for vaccination (Figure 1), which was followed by additional phases for a booster or precautionary dose, newspapers have noted that migrants were rarely placed on the national priority list for the COVID-19 vaccine [13]. However, several state governments implemented special initiatives to reach migrants. Kerala implemented policies to ensure all migrants received free vaccination [14], Tamil Nadu created a new portal for migrants to register for the vaccine [15] and invested in long-term housing to improve their living conditions [16], and Telangana and Kerala made videos in languages spoken by migrants to explain the registration process [17,18].

While these initiatives were crucial, there remained a significant need to increase vaccine uptake among migrants, particularly in states like Tamil Nadu and Punjab, where migrant populations have been disproportionately large and have required more tailored outreach mechanisms. To do this, the USAID-funded MOMENTUM Routine Immunization Transformation and Equity (the project) supported the Government of India to develop contextually appropriate local strategies. The project partnered with nongovernmental community-based organizations and provided them with financial and technical resources. (In this paper, project and community-based staff are collectively referred to as “project staff”). In turn, the project collaborated with additional local partners (health and non-health public and private actors) to design and implement program strategies to identify, reach, and vaccinate migrants in Tamil Nadu and Punjab. (These partnerships were not supported financially).

This case study answers the following questions: Who were the project partners and what were the roles of each entity? What strategies did partners implement to increase COVID-19 vaccine uptake among migrants? What were the perceptions about the effectiveness of these strategies?

Answering these questions is critical in the broader backdrop of localization, where funding agencies are increasingly prioritizing local leadership and management, particularly of the actors who are closest to the communities and best positioned to anticipate and meet their needs [25]. Given this shift, it is important to understand who the project’s partners were and their roles during the pandemic, including if and how the strategies they developed contributed to vaccine uptake among migrants. These findings can also inform ongoing discussions of leveraging local partnerships to enhance pandemic preparedness, strengthen routine immunization efforts, and build more resilient health systems, particularly in countries where local partners can support government objectives.

## 2. Materials and Methods

### 2.1. Study Context

We selected two project states, Tamil Nadu and Punjab, to understand the local partnerships established to reach migrant workers and to provide insights into these partnerships and their implemented strategies. Punjab, located in northwestern India and bordering Pakistan, is primarily agricultural and, according to the 2011 Census (the most recent available), has a population of approximately 27.7 million [26]. The state performs better than the national average on health indicators [27,28]. Tamil Nadu is the southernmost state in India and had a population of around 72 million as of 2011 [29]. It has the largest number of factories in the country [30], with nearly 50% of its population living in urban areas [31], and is known for having some of the best health outcomes in India [32,33,34].

Both states have a high influx of migrant workers from northern and eastern states like Uttar Pradesh, Bihar, Jharkhand, Chhattisgarh, Odisha, and Bengal. Tamil Nadu hosts roughly 3.49 million migrant workers [1], while Punjab has about 1.24 million migrants from other states [1]. These figures are outdated and likely underestimated since those employed in local grocery stores and eateries tend to reside without official records [35]. Table 1 summarizes the characteristics of migrant populations in the study districts and the industries in which they work.

Before the project began, 59% of eligible individuals in Tamil Nadu had completed full COVID-19 vaccination and 86% had received at least one dose [36]. To achieve these coverage levels, the government implemented initiatives including mega vaccination camps [37] and the Makkalai Thedi Maruthuvam (Doorstep Health Care) scheme, which aimed to provide medical care, increase vaccine awareness, and enhance access to preventive health services during the pandemic [38]. These initiatives supported migrant vaccination and increased first-dose coverage among the general population. When the project started in January 2022, it collaborated with the government to develop tailored strategies to increase second-dose and booster uptake, particularly among migrants. Then, the project focused on increasing second-dose uptake, followed by a focus on booster doses in May 2022, when the state had achieved relatively high first-dose coverage.

Conversely, in Punjab, in the initial phase of the project in August 2021, coverage for the first dose hovered around 45.3% and second doses hovered around 14% based on the COWIN data available at the time [39]. A government frontline health worker strike disrupted COVID-19 vaccine operations, resulting in vaccination camps being delayed or canceled [40] and a backlog of data waiting to be entered into the COWIN portal—the national system for vaccination registration and appointment scheduling, identity verification, vaccination status and certificates [41]. The project first focused on mitigating data management challenges, and then it prioritized partnership activities to increase vaccine uptake among migrants.

### 2.2. Study Design

We designed a qualitative explanatory case study guided by the Behavioral and Social Drivers (BeSD) [42] and the RE-AIM implementation science frameworks [43] to answer the outlined research questions. The first two questions—about project partners, their roles, and the strategies they developed to increase COVID-19 vaccine uptake among migrants—are descriptive in nature. We used BeSD to explain how the interventions were tailored to overcome migrants’ barriers to vaccination. To assess perceptions of the effectiveness of these strategies, we evaluated three components of the RE-AIM framework: reach, effectiveness, and implementation.

### 2.3. Data Collection

We conducted qualitative in-depth interviews (IDIs) and focus group discussions (FGDs) with five respondent categories in both states (Table 2): (1) migrant populations who were exposed to the project’s activities (e.g., received communications materials, interacted with staff and partners); (2) state-and district-level project teams; (3) partners who worked with the project team to tailor and implement interventions; (4) community leaders; and (5) district- and state-level immunization staff who supported the implementation of project interventions.

The IDIs and FGDs were led by the research team (not involved in project implementation) and conducted in Tamil, Punjabi, Hindi, or English, according to respondents’ preferences. Guides for both the FGDs and IDIs were informed by the BeSD and RE-AIM frameworks. Respondents were identified in collaboration with project staff and partners, with the aim of selecting individuals working in various industries within the states. IDIs lasted 20–30 min, and FGDs approximately 40 min. With permission, the interviews were recorded, transcribed, and translated into English. Data were collected from December 2022 to February 2023 at respondents’ worksites, or in the case of some migrants in Punjab, near their residences.

### 2.4. Data Analysis

We conducted a thematic analysis using the framework method [44]. First, we developed an analytical framework informed by BeSD and RE-AIM, consisting of the following categories: (1) internal, social, and behavioral factors influencing vaccine intention and uptake; (2) program activities and strategies designed and implemented; (3) roles of implementers and supporting actors; and (4) implementation experiences, including challenges, facilitators, and program adaptations. Four co-authors extracted all qualitative data from the IDIs and FGDs into a spreadsheet using these categories. We wrote memos summarizing information by category, comparing and contrasting findings across districts and states and respondent categories to identify cross-cutting or diverging themes. Throughout data collection and analysis, the research team, including data collectors and analysts, debriefed weekly. We presented preliminary results to selected respondents and asked for feedback to enhance the credibility and trustworthiness of our findings. Based on their feedback, we clarified the interpretation of the results.

### 2.5. Ethical Considerations

This study was conducted in accordance with the Declaration of Helsinki and approved by the institutional review boards of SIGMA Research and Consulting, New Delhi (Protocol code U74140DL2008PTC182567 and 5 November 2022) and John Snow, Inc. (22–35E PH2 and 16 September 2022). Informed consent was obtained from all study respondents.

## 3. Results

The results are organized according to the three study questions.

### 3.1. Who Were Project Partners and What Were the Roles of Each Entity?

The Tamil Nadu and Punjab Departments of Health (DOHs) were key project partners. While the government had the capacity to organize vaccine camps and provide critical resources, it needed surge capacity support to identify which migrant groups to target. The project filled this gap by facilitating partnerships that helped identify un- and under-vaccinated migrant groups and appointed community volunteers in urban areas to locate those eligible for COVID-19 vaccination. By combining their complementary skill sets and resources, the project collaborated with frontline health workers to map migrant populations in communities in Punjab and coordinated with partners in both states on the dates and locations of various vaccination camps. In turn, the DOHs provided vaccinators and vaccines.

In Tamil Nadu, the project was built on a government-run partnership between the Departments of Labor and Health that sent mobile medical units (MMUs) to serve construction workers in Madurai. The Department of Labor provided information about major construction sites and the DOH scheduled visits, during which MMUs provided primary care services including screening for infectious and non-communicable diseases, and the project provided COVID-19 vaccine counselors and vaccinators. The project also invited MMU staff to conduct health screenings in urban communities where new migrants were identified.

The project facilitated partnerships in both states between the DOHs and private employers, such as construction, agriculture, fertilizer, and brick kiln companies, and with roadside vendors and community-based organizations. For example, in Tamil Nadu, Rotary Club members helped connect the project to local hotels that hired migrants. After establishing partnerships, the project explained its objective, coordinated with the human resources department to list migrants eligible for vaccination, and organized camps at workplaces and residences in collaboration with the DOH. After vaccination camps, project staff maintained contact with partners to follow up on the vaccination status of new migrants and those who chose to remain unvaccinated.

In Punjab, where religion influences the broader social culture, the project partnered with religious institutions that migrants visited for free food. The project held vaccination sessions in these institutions and developed videos of religious leaders describing the importance of vaccination. The project facilitated a partnership between the Border Security Force and the DOH to transport government frontline health workers to vaccinate migrants in communities near the Pakistan border. Table 3 summarizes the roles of the project and its partners.

### 3.2. What Strategies Did Partners Implement to Improve COVID-19 Vaccine Uptake Among Migrants?

The findings below are grouped according to the three BeSD categories [45].

#### 3.2.1. Thinking and Feeling

Many migrants were hesitant to get vaccinated due to deep-seated concerns about safety, particularly after the death of a famous actor in Tamil Nadu shortly after vaccination. In the Amritsar and Ludhiana districts of Punjab, some migrants held misconceptions that the vaccine was a cover for population control or that COVID-19 primarily affected affluent individuals, as one migrant remarked: “Hard-working people will never get corona. This is the government’s propaganda” (FGD-02, migrant worker, Punjab).

Similarly, a migrant tied his understanding of contracting COVID-19 to a person’s work habits:

*“The people who are working will never be infected from corona, the ones who are idly sitting at home are more prone to be infected by corona. The people who are hardworking their sweat comes out. People have to work and when they get sweat corona infection will not affect their body”*

(FGD-09, migrant worker, Tamil Nadu).

The project developed videos and flyers in languages spoken by migrants summarizing the benefits and safety of COVID-19 vaccines to dispel fears, myths, and misconceptions about vaccination and to highlight its importance. In addition, before the vaccination camps, the project held discussions with migrants to allay concerns about vaccines and discredit commonly held misconceptions. Video messages about COVID-19 featuring religious leaders were shown at religious sites, and frontline health workers frequently showed them during visits to urban migrant settlements. Beyond developing materials, project staff periodically followed up with un- and under-vaccinated migrant workers by visiting their worksites or residences with government frontline health workers.

#### 3.2.2. Social Processes

Social and peer pressure significantly influenced how migrants perceived their vaccine risk and decision to get vaccinated. Migrants who had received a prior vaccine dose were encouraged by peers and family members and reassured when they saw friends vaccinated without side effects. A couple of migrants were motivated by government leaders who endorsed the vaccine. One stated, “I heard on the radio that the prime minister was talking about vaccination; he is the head of our state, so I too decided to get vaccinated” (FGD-08, migrant worker, Punjab).

However, a lack of trusted voices in brick kiln and construction workers’ immediate network in both states fueled vaccine fear and misinformation. In response, the project engaged three key types of workplace influencers: safety or welfare officers responsible for worker safety and access to government benefits; contractors who hire migrants and are often from the same village or state; and company owners. These influencers were encouraged to emphasize the importance of vaccination as conveyed by project staff and frontline health workers during vaccination camp discussions and to receive the vaccine in front of employees to build confidence in its safety. They also had a crucial role in gathering information on migrant vaccination status and maintaining lists of those due for vaccination. In Punjab, where religious leaders were generally trusted, they reinforced the importance of vaccines through video messages and during vaccination camps. These leaders also received the vaccine at the camps.

Migrants who had received a prior vaccine dose were motivated to get the second dose so they could obtain a vaccine certificate that allowed them to cross state lines and travel for work. Moreover, most private employers of migrants in Tamil Nadu and Punjab enforced the COVID-19 vaccination policy. Specifically, the human resources team or equivalent person at private industries typically passed an order that all workers needed to receive a complete primary series of the COVID-19 vaccines before returning to work.

#### 3.2.3. Practical Issues

Migrants faced multiple practical challenges to COVID-19 vaccination, including (1) difficulties retrieving vaccination records, as government health workers struggled to access information about the number of doses and vaccine types received; (2) a lack of vaccine certificates and text messages containing vaccine information due to frequently changing phone numbers; and (3) language barriers, as many migrants from Hindi-speaking states have limited knowledge of Tamil, making it difficult to ask for and receive important information from government health staff, who often do not speak Hindi.

To mitigate these challenges and concerns about lost wages, vaccination camps were set up at workplaces in Tamil Nadu, making it easier for private employers to enforce their COVID-19 vaccination policies. In Punjab, where many laborers live in urban settlements near their workplaces, camps were arranged in early morning and late evening to accommodate work schedules. The project team organized vaccination drives during religious festivals and at religious institutions that migrants frequented.

**Figure 2:**
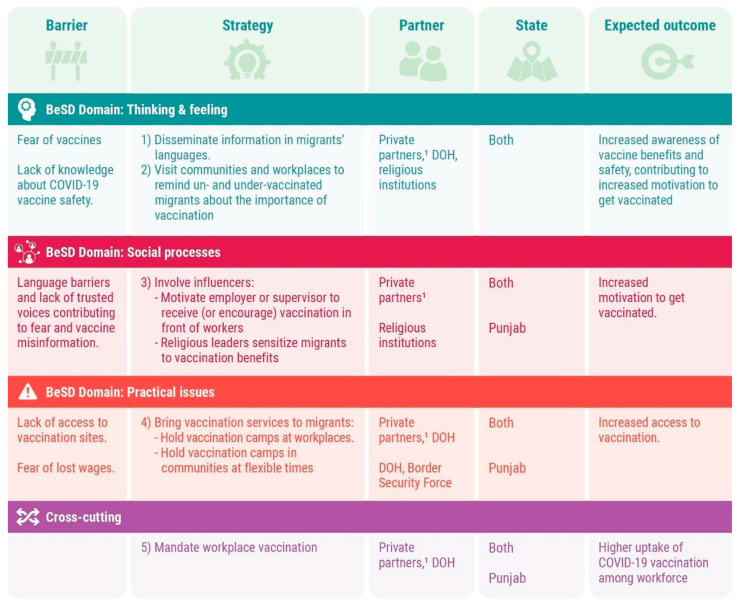
Strategies developed by partners to increase COVID-19 vaccine uptake among migrants. ^1^ Employers of brick kilns, construction sites, hotels, and fertilizer and agricultural companies.

Similarly, to increase access to vaccination points for migrant communities on the Pakistan–Punjab border, the Border Security Force provided transportation for frontline health workers to access and establish relationships with migrants who were unvaccinated. During these visits, frontline health workers used participatory approaches such as games, skits, and community dialogues to educate mothers and their children about the benefits of COVID-19 vaccines and create a more comfortable environment to encourage their participation. Figure 2 summarizes the strategies developed to overcome barriers and leverage motivators for COVID-19 vaccine uptake.

### 3.3. What Were the Perceptions About the Effectiveness of These Strategies?

Interviews with migrants in Tamil Nadu and Punjab revealed several strategies (below) that shifted their perception of and motivated them to get vaccinated and made it easier for them to do so.

#### 3.3.1. Disseminate Information in Migrants’ Languages

Although disseminating informational materials and videos in migrants’ languages was a key strategy, our interviews with key informants provided no evidence that this approach was effective on its own. This strategy was combined with others, such as asking influencers to hand out materials before vaccination camps or having project staff do so during workplace visits in Tamil Nadu and Punjab and in migrant communities in Punjab.

#### 3.3.2. Ensure Frequent On-Site Visits

Migrants reported that frequent on-site visits by project staff to monitor the vaccination status of under- and unvaccinated migrant workers provided continuous opportunities to ask questions and learn about the vaccine. These visits and the workplace vaccination camps made getting vaccinated easier and contributed to increased uptake. A migrant who temporarily moved to Madurai to work in a brick kiln described the benefits of this strategy:

*“Our area nurse and local body members approached and advised to take vaccine in the camp conducted in the village. But due to fear of side effect and considering loss of wage and no one to do daily household chores I didn’t take the vaccine. After some time a staff [project staff member] approached regularly again and again and created awareness and provide confidence that there won’t be any side effect and can-do work after vaccination. The staff brought the vaccine to the worksite, and I took it”.*

[FGD 05, migrant worker, Tamil Nadu]

#### 3.3.3. Involve Influencers

Involving a range of community influencers raised vaccine awareness and increased migrants’ confidence in its safety and necessity. In Punjab, religious leaders guided staff in slums and villages to raise vaccine awareness among migrant workers. As one affiliate manager at a religious institution said:

*“The manager of a gurdwara has 20 subordinates under him from different villages. After getting awareness and importance of vaccines…they in turn disseminate the messages in their respective villages and support the project teams to organize camps and persuade people to go for vaccination. The pamphlets (notice) of the vaccination camp used to be put at the notice board of the gurdwara and people who come to the gurdwara also see and get benefits from the camp”.*

[IDI-02, project partner, Punjab]

Partners said that the project involvement of influencers increased migrants’ awareness of the value of vaccinations. Construction workers in Tamil Nadu trusted safety/welfare officers because they spoke the same language and paid close attention to the workers’ safety and needs. Both migrants and safety officers suggested that this trust significantly influenced migrants’ vaccination decisions. One safety/welfare officer said the following:

*“I used to arrange training and conduct meetings to discuss their problems. Further, I used to be in touch with them continuously and since I am helping to solve their issues that made them listen and accept vaccines”.*

[IDI-7, project partner, Punjab]

Similarly, contractors were described as enforcers of the compulsory vaccine policies and helped migrants obtain vaccination certificates. One MMU medical officer said the following:

*“There will be a contractor for 10–20 people, and all will listen to his say. Each migrant group has one such contractor who is a major influencer”.*

[IDI-05, government staff, Tamil Nadu]

Seeing a company owner receive the vaccine directly motivated migrants to do so as well. While this helped some workers see proof of safety, in some cases, it may have created pressure to follow suit. One migrant factory worker described how the owner’s actions influenced his decision:

*“If the owner takes the vaccine, we would also take it. For example, if the house owner asks us to leave the house, we must leave. In the same way we also accepted to take vaccine when our owner informed”.*

[IDI-14, migrant worker, Tamil Nadu]

#### 3.3.4. Bring Vaccination Services to Migrants’ Homes/Workplaces

Migrants and employers described the additional benefits of vaccination at workplaces, such as being able to promptly return to work and the elimination of practical challenges to offsite vaccination such as potential wage loss and transportation costs. A human resources manager in Punjab indicated that these factors contributed to all migrant workers getting vaccinated:

*“On-site vaccination camp by the project team made 60 migrants from Bihar working in our fertilizer company to get vaccinated”.*

[IDI- 15, project partner, Tamil Nadu]

Bringing vaccination to migrant communities in Punjab also made a difference, as one migrant worker noted:

*“Vaccination camp organized in our area where we are living was good because I did not have to walk long distances and I didn’t lose my day wages. The staff visited each household and motivated people to come and get vaccination. Because of the camp each person got vaccinated, if it was not organized in our area, not everybody would have got vaccinated”.*

[FGD, migrant worker, Punjab]

#### 3.3.5. Mandate Vaccination

Construction workers in both states indicated that mandating COVID-19 vaccination increased uptake. Migrant workers and their supervisors indicated that the mandatory vaccination created urgency to get the vaccine and certificate, without which migrants would be unable to earn a livelihood.

*“Here the manager would inform everyone to take the injection. If they [workers] do not come to work also, they will give them a phone call and ask them to come here and take the injection. People can come for work only after getting the injection, otherwise no, and that is why everyone fully took the injection”.*

[FGD-1, migrant worker, Punjab].

Another human resource manager in a private company described policy enforcement.

*“They [employees] come and show me the certificate. If not, we will tell them that it is government rule, whichever construction site you go, COVID certificate is mandatory. Otherwise, they [company owner] won’t allow you”.*

[IDI-16, project partner, Tamil Nadu]

### 3.4. Facilitators and Barriers to the Implementation of These Strategies

This section outlines cross-cutting vaccine facilitators and barriers that do not align with a specific strategy. In terms of facilitators, the Tamil Nadu State government demonstrated strong political will at all levels of the health system. This was evident in its policies promoting migrant health, such as the implementation of MMUs. This commitment to migrant health also supported the implementation of project strategies, particularly for on-site vaccination efforts targeting migrants who lacked the proper identification needed to receive the vaccine.

At the district level, government task forces adapted the Government of India’s instructions on vaccinating individuals without proper identification. They established clear protocols and delegated the responsibility for migrants’ vaccination to the appropriate personnel within the health care system. This coordination within the DOHs advanced the project and government goal to improve migrant health. One state official reflected on coordination within the DOHs:

*“So, even those things were covered with the deputy director, health services, and in coordination with the district task force committee meetings. So, a district task force meeting will be convened and for those people without identity, a list of these people would be given and the caretaker or the in charge of the hospital will be then given the charge to get them vaccinated”.*

[IDI-14, state government official, Tamil Nadu]

According to migrants, the project’s strategy of hiring volunteers from the urban residential areas in which the migrants live built trust and rapport; it created an awareness of new migrants and reinforced linkages with health workers who served communities, facilitating vaccination camp coordination.

Despite these successes, state- and district-level project staff said that the initial stages of the project were challenging. In Punjab, for example, it was necessary to overcome basic health system barriers, such as entering backlog data for the first dose administered by the government before starting project activities. Both states needed to build relationships with frontline workers; define and agree on processes for collaboration and coordination for vaccination camps; and manage logistics related to vaccinators and vaccine supply at the block- and district- levels of the health system.

## 4. Discussion

This study presents insights into identifying, reaching, and supporting vaccination uptake among migrant populations through diverse partnerships. We examined the roles of partners and the strategies developed in collaboration with the DOHs and non-health sectors—such as private sector employers, religious institutions, and governmental agencies like the Department of Labor and the Border Security Force—to increase vaccine awareness and uptake among migrant populations in Tamil Nadu and Punjab. The study findings build on previous findings that show the importance of partnering with non-health sectors [46,47,48].

First, collaboration with community-based partners like Rotary International, the private sector, and religious institutions, alongside government health and non-health sectors, were instrumental in mapping and reaching migrant populations eligible for the COVID-19 vaccine. The partnership with the Border Security Force enabled frontline health workers to access border migrants, while religious leaders extended their influence to slums and villages where migrants reside. Collaboration with human resources teams and on-site managers in private sector companies increased awareness and reinforced the importance of vaccination.

Second, strategies such as organizing vaccination camps at workplaces and residences reduced practical barriers to uptake among migrants. These findings are similar to studies that have found that offering vaccination at workplace increases coverage, especially when combined with employer reminders [49] and broader advocacy efforts [50]. Findings from low- and middle-income countries also suggest that for vulnerable groups like tribal populations, bringing vaccination closer to communities and offering them flexible times, as the project did in Punjab, facilitates uptake [51,52].

For certain migrants, the on-site issuance of vaccination certificates following workplace vaccination and compulsory vaccination policies were strong motivators for getting vaccinated. Other studies suggest that mandating vaccination may be associated with vaccine uptake for certain, but not all, groups [53]. In this project, migrants viewed vaccine certificates as allowing them to sustain a livelihood.

Third, our study highlights particular barriers that migrants face: they move to cities largely to work in unorganized sectors and have job insecurity, language and cultural differences, and limited social networks. Aligned with previous research [54], we found these factors limited their knowledge of and access to health care services, including vaccination. Without established networks, many migrants relied on trusted workplace connections, including human resource managers, contractors, and welfare officers, to influence their vaccination decisions. This trust was rooted not only in these individuals’ responsibilities but also in their ability to communicate in the same language as the migrants. This aligns with another study that found benefits when trusted people conveyed information about COVID-19 vaccination to migrants in their own language [55].

Trust was further demonstrated by the participation of industry and company owners, particularly when they received vaccines in front of workers. This helped establish trust and encouraged vaccine uptake among the workforce. Previous studies have similarly underscored the importance of involving trust influencers such as religious and local leaders and civil society organizations in activities to enhance vaccination rates among migrants [56,57]. Finally, the project’s partnerships facilitated the development of unique, tailored strategies to increase vaccine uptake that could only be achieved through such diverse collaborations. The DOH expanded its vaccination camps to non-traditional locations, such as workplaces and religious institutions, through project-facilitated partnerships. These partnerships gave the DOHs and the government access to the workplace influencers and religious and community leaders who shaped migrants’ decision-making and reinforced their messages on vaccine awareness and safety. The involvement of multiple actors enabled a more cohesive approach to increasing vaccine awareness and uptake, similar to other findings [50]. These diverse collaborations also highlight the importance of context-specific partnerships in shaping vaccine uptake strategies. For instance, trust in different actors, such as the Border Security Force—perceived as a trusted source for vaccination programs in the context of our study—may vary and may not be applicable to other contexts within India or other countries due to varying sociocultural and political factors.

The strategies and partnerships identified in this study have broader implications for future public health interventions. The study demonstrates that the private sector and faith-based organizations can be employed in multiple health areas such as routine immunization. Moreover, these partnerships will be most effective when they are localized to organizations who know the communities best and design strategies to respond directly to identified barriers and motivators. For example, trusted network members and influencers identified through partnerships could enhance migrants’ routine immunization uptake, access to primary health care services, and use of health programs. The barriers faced by migrants can also be overcome by designing and implementing strategies such as appointing health workers fluent in migrants’ languages to increase trust and health care service access. Furthermore, the success of workplace vaccination strategies suggests that similar approaches could be effective for routine adult vaccination initiatives. These strategies could be adapted for childhood vaccination, particularly those that bring vaccinations closer to migrants’ residences, where children are likely to be present.

### Limitations

First, we were unable to interview all project partners due to varying availability, but we made efforts to capture diverse perspectives by interviewing partners and state- and district-level immunization staff who worked closely with them. Second, conducting some interviews at worksites may have introduced bias because migrants’ supervisors were nearby. We attempted to mitigate this by finding quiet places away from supervisors. Third, various factors influence an individual’s vaccine uptake, and it was not possible to disentangle their respective contributions. Fourth, it is important to note that most of our findings predominantly reflect perspectives from male migrants, which may not fully represent the experiences and perspectives of female migrants. Future studies should aim to assess the relative importance of different barriers and motivators to develop vaccination-demand programs.

## 5. Conclusions

This study highlights the effectiveness of strategic partnerships between health and non-health sectors in supporting vaccine uptake among migrants in Tamil Nadu and Punjab. Collaboration with the DOHs, private sector employers, religious institutions, and non-health-focused government agencies were found to expand access to COVID-19 vaccination through on-site camps and trusted influencers. These efforts mitigated barriers related to job insecurity and language differences, indicating that similar partnership strategies could strengthen routine immunization and other public health initiatives for migrant populations.

## Figures and Tables

**Table 1:** Characteristics of migrants in study districts.

State	District	Industry (State of Origin)	Characteristics
Tamil Nadu	Madurai	Construction (Uttar Pradesh, Bihar) Fertilizer (Odisha) Roadside vendors (e.g., toymakers, and artisans) (Rajasthan) Brick kiln & matchstick factories (rural villages in Tamil Nadu)	These migrants came to Tamil Nadu temporarily for work and lived in rented rooms near the worksite or on company premises. Migrants stayed for years but went home for major life events (weddings, births, deaths). They lived in makeshift roadside dwellings. These migrants were seasonal workers who lived in makeshift dwellings on company premises.
Punjab	Ludhiana	Textiles (West Bengal, Orissa) Manual labor (e.g., (Uttar Pradesh, Bihar and Rajasthan)	Migrants across these industries live in slum communities close to the place of work. Some of these migrants are second-generation.
	Bhatinda Amritsar	Construction (Bihar, Jharkhand) Construction (Bihar, Jharkhand) Brick-kiln factories (Uttar Pradesh, Rajasthan, Punjab)	

**Table 2:** FGDs and IDIs, by respondent category and state.

Respondent	Tamil Nadu	Punjab	Total
Migrants			
FGD	15	8	23
IDI	14	-	14
Project team			
FGD	2	6	8
Project partners			
IDI	3	5	8
Community leaders			
IDI	4	10	14
District-level government immunization staff			
IDI	4	4	8
State-level government immunization staff			
IDI	4	2	6
Total	17 FGDs, 29 IDIs	14 FGDs, 21 IDIs	31 FGDs, 50 IDIs

**Table 3:** Partner roles in increasing COVID-19 vaccine uptake among migrants.

Partner	Partner Role	Project Role ^1^
***Both states***		
DOH	• Identified migrants through frontline health workers. • Set up vaccination camps with the project. • Provided vaccinators and data entry staff at vaccination camps.	• Hired volunteers from the community/block to map new arrivals eligible for vaccination. • Met weekly with the village health nurse. • Supplied lists of individuals eligible for vaccination. • Coordinated day/time/location for vaccination camps ^2^.
Community-based organizations & private partners	• Connected the project with additional partners to reach missed migrants.	• Followed up with identified partners.
***Tamil Nadu***		
Department of Labor and DOH	• The Department of Labor shared information about major construction sites. • The DOH held MMUs to conduct health screenings and provide health counseling at the sites. Sometimes DOH provided a vaccinator.	• Counseled construction workers about the importance of and provided vaccination through the project-appointed or government vaccinator.
***Punjab***		
Religious institutions	• Coordinated vaccination sessions in gurudwaras (places of worship). • Conveyed vaccination messages.	• Developed informational material in various languages.
Border Security Force	• Transported frontline health workers to vaccinate migrants near the Pakistan border.	• Coordinated with the DOH on vaccination location and follow up.

^1^ Community-based organizations that were the project implementing partners and worked with project staff. ^2^ In Tamil Nadu, some vaccination camps included project-appointed vaccinators.

## Data Availability

The data presented in the study are available on request from the corresponding author. The data are not publicly available due to the privacy concerns of the respondents.
